# Anti-BNLF2b antibody for accurate diagnosis of nasopharyngeal carcinoma: a prospective comparative study

**DOI:** 10.1186/s12885-026-16523-z

**Published:** 2026-07-21

**Authors:** Bixia Huang, Lu Yang, Xingxing Luo, Weiliang Dai, Huoqiang Chen, Zhanze Chen

**Affiliations:** 1 Department of Clinical Laboratory, School of Medicine, The First People’s Hospital of Foshan (Foshan Hospital, Southern University of Science and Technology), Southern University of Science and Technology, Foshan, Guangdong China; 2 Foshan Key Laboratory of Skin Tissue Engineering and Precision Diagnosis and Treatment of Infectious Skin Diseases, Foshan, Guangdong China; 3 Beijing Wantai Biological Pharmacy Enterprise Co., Ltd, Beijing, China

**Keywords:** Nasopharyngeal carcinoma, Epstein-Barr virus, Anti-BNLF2b antibody, Serological biomarkers, Diagnosis

## Abstract

**Background:**

Nasopharyngeal carcinoma (NPC) is highly prevalent in Southern China and Southeast Asia and early detection significantly improves prognosis. Conventional Epstein-Barr virus (EBV) serological markers VCA-IgA and EBNA1-IgA have been widely used for NPC screening yet their performance remains unsatisfactory in real-world screening scenarios. Anti-BNLF2b antibody (P85-Ab) has emerged as a promising novel serological biomarker. However, comprehensive comparisons with conventional markers in clinically diverse cohorts incorporating diverse non-NPC patients remain insufficiently evaluated.

**Methods:**

A prospective case-control study was performed, recruiting 122 NPC patients and 185 control subjects (99 healthy individuals and 86 patients with non-NPC disorders). Six serological EBV antibody biomarkers were evaluated: Wantai_P85-Ab, Wantai_EBNA1-IgA, Wantai_VCA-IgA, Snibe_VCA-IgA, Wantai_Zta-IgA, and Tarcine_Rta-IgG. Propensity score matching (PSM) was implemented to balance demographic covariates between NPC cases and controls. Receiver operating characteristic (ROC) curve analysis was adopted to calculate the area under curve (AUC), sensitivity and specificity for evaluating diagnostic accuracy, and concordance analysis was conducted to explore the complement value of distinct biomarkers.

**Results:**

After PSM, the matched analytical cohort contained 244 participants (122 NPC cases, 122 controls). Wantai_P85-Ab achieved an AUC of 0.989 (95% CI: 0.966–0.998), which was significantly higher than Wantai_EBNA1-IgA (0.942, 95% CI: 0.904–0.967), Wantai_VCA-IgA (0.874, 95% CI: 0.826–0.913), Wantai_Zta-IgA (0.847, 95% CI: 0.796–0.890), Tarcine_Rta-IgG (0.774, 95% CI: 0.716–0.824), and Snibe_VCA-IgA (0.601, 95% CI: 0.536–0.663) (all *P* < 0.001). Wantai_P85-Ab achieved a sensitivity of 94.26% (95% CI: 88.63-97.19) and specificity of 98.36% (95% CI: 94.22-99.55). Subgroup specificity analysis showed P85-Ab reached 100.00% (95% CI: 96.26-100.00) when distinguishing NPC from healthy controls, and 95.35% (95% CI: 88.64-98.18) against non-NPC disease patients. Furthermore, Wantai_P85-Ab exhibited comparable sensitivity in early-stage (*n* = 20) NPC (95.00%, 95% CI: 76.39–99.11) and advanced-stage (*n* = 102) NPC (94.12%, 95% CI: 87.76–97.28). Parallel combination of Wantai_P85-Ab and Wantai_EBNA1-IgA modestly increased sensitivity to 97.54% (95% CI: 93.02-99.16) with a significant declined specificity of 92.62% (95% CI: 86.57-96.07).

**Conclusions:**

This prospective comparative study with mixed clinical controls indicates that Wantai_P85-Ab has potential advantages over conventional single EBV serological markers. These findings provide supplementary clinical evidence supporting the application of P85-Ab as a primary serological biomarker for NPC screening and auxiliary diagnosis in endemic regions.

**Supplementary Information:**

The online version contains supplementary material available at https://doi.org/10.1186/s12885-026-16523-z.

## Introduction

Nasopharyngeal carcinoma (NPC) is a malignant epithelial tumor originating from the mucosal lining of the nasopharynx. Global surveillance data from 2022 recorded approximately 120,000 incident cases and 73,000 fatalities, with a pronounced geographic concentration in East and Southeast Asia, particularly Southern China, which accounts for over 80% of the global burden [1]. Nearly 70% of patients are diagnosed with advanced-stage disease, leaving limited curative therapeutic choices. Despite the use of multimodality regimens combining radiotherapy, chemotherapy, and surgery, the 5-year survival rate for advanced-stage disease (51.4%-76.0%) remains markedly lower than that for early-stage disease (82.6%-86.6%), highlighting the pivotal role of early diagnosis in improving patient outcomes [2–5]. The NPC involves a complex interplay of Epstein-Barr virus (EBV) infection, genetic susceptibility, and environmental factors [6]. Furthermore, conceptualizing NPC as an ecological disease suggests that its development and progression can be dynamically understood from ecological and evolutionary perspectives [7]. Within this dynamic host-virus interaction, EBV plays a central role in NPC tumorigenesis, establishing latent infection in nasopharyngeal epithelial cells and contributing to their malignant progression [8].

Early serological screening, introduced in the late 1970s, relied on immunofluorescence assay to detect IgA against the EBV viral capsid antigen (VCA-IgA) and early antigen (EA-IgA) [9], but its limited sensitivity (~ 50.9%) and poor inter-laboratory reproducibility constrained large-scale application [10]. The shift to enzyme-linked immunosorbent assay (ELISA) enabled standardized, quantitative measurement across a broader antibody panel [11, 12]. A dual-marker ELISA combining VCA-IgA with EBNA1-IgA raised sensitivity to 75.0% while maintaining high specificity (~ 98.5%), establishing it as the reference standard for population-level NPC screening [13], and a large cluster-randomized controlled trial further showed that screening guided by this combination reduced NPC-attributable mortality by 30% over 12 years [14]. A meta-analysis summarized the performance of individual markers: VCA-IgA reached 87% sensitivity and 93% specificity, EBNA1-IgA 83% and 96%, while Zta-IgA and Rta-IgG each showed lower sensitivity (~ 70–75%) with more heterogeneous specificity [15]. Nonetheless, the sensitivity and positive predictive value (PPV, ~ 4.3% for the VCA-IgA/EBNA1-IgA model) remains inadequate to meet the demands of NPC screening and early detection, underscoring the need for more sensitive and specific diagnostic tools [16].

In a landmark prospective screening study of 24,852 participants in Southern China, the newly identified serological anti-BNLF2b antibody (P85-Ab) demonstrated a sensitivity of 97.9%, a specificity of 98.3%, and a positive predictive value of 10.0%-approximately twice that of the conventional two-antibody method [16]. This robust performance established P85-Ab as a top-performing biomarker in NPC screening, which had not been ranked among the leading candidates in earlier serological profiling studies due to differences in the antigens and detection systems [17, 18]. Independent validation studies conducted across geographically and epidemiologically distinct regions of China have corroborated the strong discriminative ability of P85-Ab (AUCs ranging from 0.949 to 0.990) [19–22]. Notably, P85-Ab achieved the highest sensitivity for early-stage disease (~ 93%), outperforming both the VCA-IgA/EBNA1-IgA antibody score and the circulating EBV DNA assay (both ~ 87%), and maintained consistent performance across age, sex, region, ethnicity, family history, and smoking subgroups, supporting its robustness across diverse populations [23]. Furthermore, the clinical utility of P85-Ab in outpatient diagnosis has been validated by a prospective cohort study of 3,777 patients with suspected NPC, which demonstrated that P85-Ab achieved a sensitivity of 93.0% and specificity of 97.3%, outperforming conventional VCA-IgA, EA-IgA, and EBNA1-IgA assays in differential diagnosis. Notably, P85-Ab maintained high sensitivity (92.0%) among asymptomatic individuals and those with non-specific symptoms. For patients presenting with NPC-specific symptoms, a triplet-antibody strategy combining P85-Ab with VCA-IgA and EBNA1-IgA further improved sensitivity to 95.9% [24]. Taken together, these findings suggest that P85-Ab holds valuable potential for both screening populations at high risk for NPC and serving as an adjunctive clinical diagnostic tool.

To provide complementary evidence in an independent population, we conducted a prospective case-control study in Foshan, China. We comprehensively evaluated P85-Ab against other conventional EBV serological markers. This broad panel enabled robust individual benchmarking and the exploration of optimal multi-marker combinations. Furthermore, to better reflect real-world clinical diagnostic challenges, our control group incorporated not only healthy individuals but also patients with non-NPC conditions, including both benign diseases and other malignancies.

## Methods

### Study population

This prospective case-control study was conducted at the First People’s Hospital of Foshan between January and December 2025 and is reported in accordance with the STARD statement for diagnosis studies [25]. Consecutive individuals with newly diagnosed NPC at initial evaluation were screened for eligibility. Final diagnosis was established by pathological examination, with clear stage information, which was assessed according to the 8th American Joint Committee on Cancer TNM Staging System [26]. Non-NPC other diseases (OD) group were collected from the Otorhinolaryngology (ENT) Clinic and Ward. The OD group included inflammatory and infectious diseases, hematologic malignancies, autoimmune diseases, neuroendocrine/neuroectodermal tumors, mesenchymal tumors, epithelial malignancies, and benign epithelial tumors. To further evaluate the differential diagnostic performance of these serological EBV antibodies, an additional dataset of 11 non-NPC epithelial tumors was collected (see Table S3 for details). Among all 97 non-NPC subjects, 66 cases (68.04%) presented with NPC-specific symptoms, including nasal obstruction, nasal discharge, epistaxis, tinnitus, hearing impairment, facial numbness, and an unexplained lump in the neck; 23 cases (23.71%) had non-specific symptoms (e.g., hoarseness, sore throat, limb numbness, fatigue, or facial rash); and 8 cases (8.25%) were asymptomatic individuals with incidentally detected positive EBV serology (*n* = 6) or a family history of NPC (*n* = 2). Healthy controls (HC) were recruited from individuals undergoing routine health examinations in our hospital during the same period. Their inclusion criteria required no nasopharyngeal suspicious symptoms including persistent epistaxis, nasal obstruction or tinnitus within the preceding 6 months.

Inclusion criteria for NPC cases and non-NPC other diseases were as follows: the samples should be collected before therapy, suspected subjects were required to have undergone endoscopy or pathological evaluation to exclude NPC. Exclusion criteria were prior malignancy treated with systemic anti-tumor therapy 3 years before blood draw and poor-quality specimens with severe hemolysis, deterioration, lipemia/chylous appearance, or obvious microbial contamination.

Complete clinical information such as age and sex was recorded for all subjects. The study was approved by the Medical Ethics Committee of the First People’s Hospital of Foshan City (Approval no. 2025-022), and all participants provided written informed consent. The study was conducted in accordance with the Declaration of Helsinki.

### Sample collection and serological EBV antibodies detection

For all participants only the earliest sample from each participant was retained if multiple samples were available. Serum had to be separated within 4 h of collection and stored at -80 °C until analysis. BNLF2b antibody (P85_Ab), VCA-IgA, EBNA1-IgA and Zta_IgA were detected using Chemiluminescent Microparticle Immunoassay (CMIA) kit (WANTAI BioPharm, Beijing, China) with automatic CMIA analyser Wan200+ (Xiamen UMIC Medical Instrument Co. Ltd, Xiamen, China) according to the manufacturers’ instructions. Rta-IgG levels were assessed using enzyme-linked immunosorbent assays (ELISA) with respective antibody detection kits provided by Tracine BioMed Inc. (Beijing, China). For the above EBV antibodies, the sample with cut-off index (COI) ≥ 1 was considered as positive; otherwise, the sample was considered as negative. VCA-IgA was measured using CMIA with antibody detection kits provided by Snibe BioMed Co., Ltd. (Shenzhen, China). Samples with concentration of 4.0 AU/ml or greater were considered VCA-IgA positive; all others were considered negative. In the parallel testing strategy, “OR” indicates that the combination is considered positive if at least one of the component markers yields a positive result. 

### Statistical analysis

All statistical analyses were performed using the GraphPad Prism 10.4.0 (GraphPad Software, Inc., SanDiego, CA, USA) software and MedCalc 22.001 (MedCalc Software Ltd. Belgium.). Continuous variables were assessed for normality using the Shapiro-Wilk test and are presented as median and interquartile range (IQR) because distributions were non-normal. The statistical comparisons between groups were performed using the Mann-Whitney U test (for two-group comparisons). Categorical variables are presented as numbers (percentages) and were compared using the chi-square test or Fisher’s exact test, as appropriate.

Diagnostic performance was evaluated using receiver operating characteristic (ROC) curves and the area under the curve (AUC) with 95% confidence interval (CI). The DeLong test was used for pairwise AUC comparisons. Sensitivity and specificity were calculated against the reference standard diagnosis, and their 95% CIs were estimated with the Wilson score method. Differences in paired sensitivities and specificities were compared using McNemar’s exact test, and 95% CIs for paired differences were calculated using the Newcombe-Wilson method. All tests were two-sided, and *P* < 0.05 was considered statistically significant.

Because age and sex differed between the NPC and non-NPC groups before matching, propensity score matching (PSM) was prespecified to reduce confounding. Propensity scores were estimated with logistic regression including age and sex as covariates. One-to-one nearest-neighbor matching without replacement was performed using the MatchIt package (version 4.2.0) in R (version 4.5.2) [27]. The caliper width was set at 2.1 times the standard deviation of the propensity score. Covariate balance was assessed using standardized mean differences (SMDs), with absolute SMD < 0.1 indicating adequate balance. The matched cohort (122 NPC cases and 122 controls) served as the primary analysis set. Diagnostic performance in the original unmatched cohort (*n* = 307) was evaluated as a prespecified sensitivity analysis.

## Results

### Baseline characteristics

A total of 307 subjects were enrolled in the initial cohort, comprising 122 NPC patients and 185 non-NPC controls. As shown in Table S1, significant demographic imbalances were identified between the two groups prior to matching, with the NPC group being older and having a higher proportion of male subjects (both SMD > 0.1). Following PSM, 244 subjects (122 per group) were retained, and all matching variables achieved satisfactory balance, with SMD values of 0.017 for sex and 0.040 for age, providing a well-balanced analytical framework for the subsequent assessment of EBV antibody diagnostic performance.

### Diagnostic performance of individual EBV antibody biomarkers

**Fig. 1:**
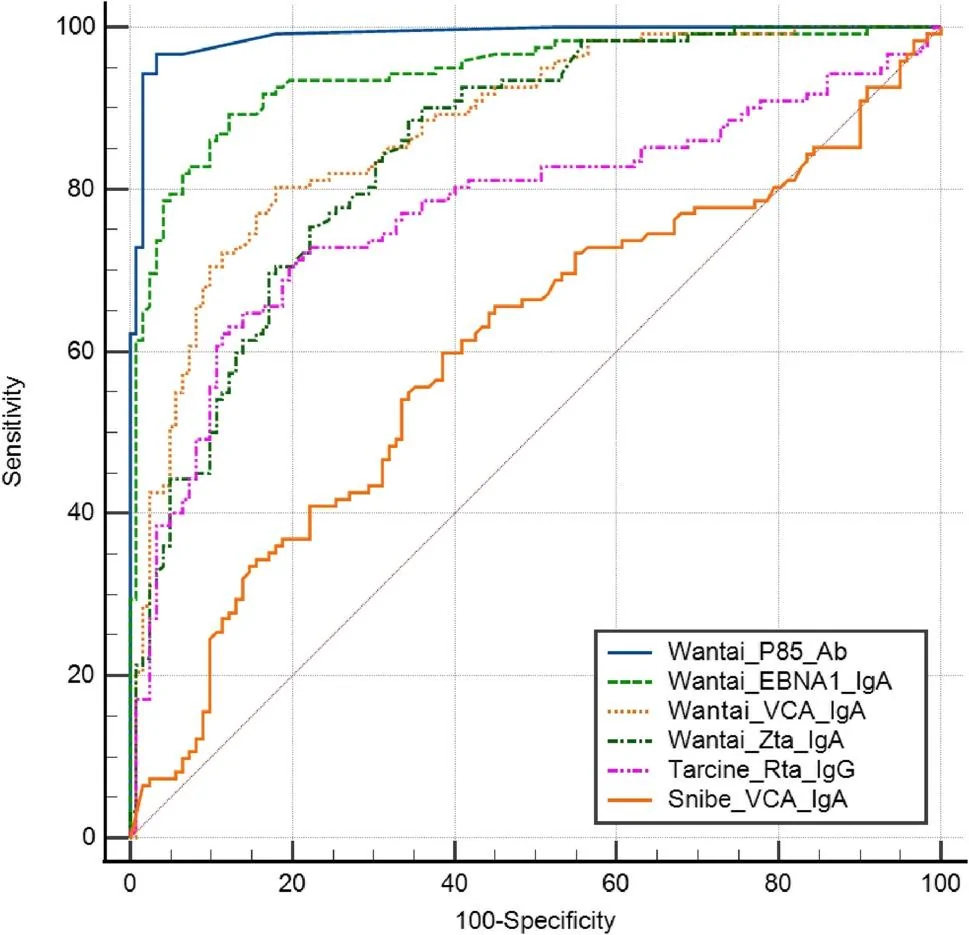
ROC curves of EBV antibody biomarkers for distinguishing nasopharyngeal carcinoma (NPC) from non-NPC subjects following propensity score matching

**Fig. 2:**
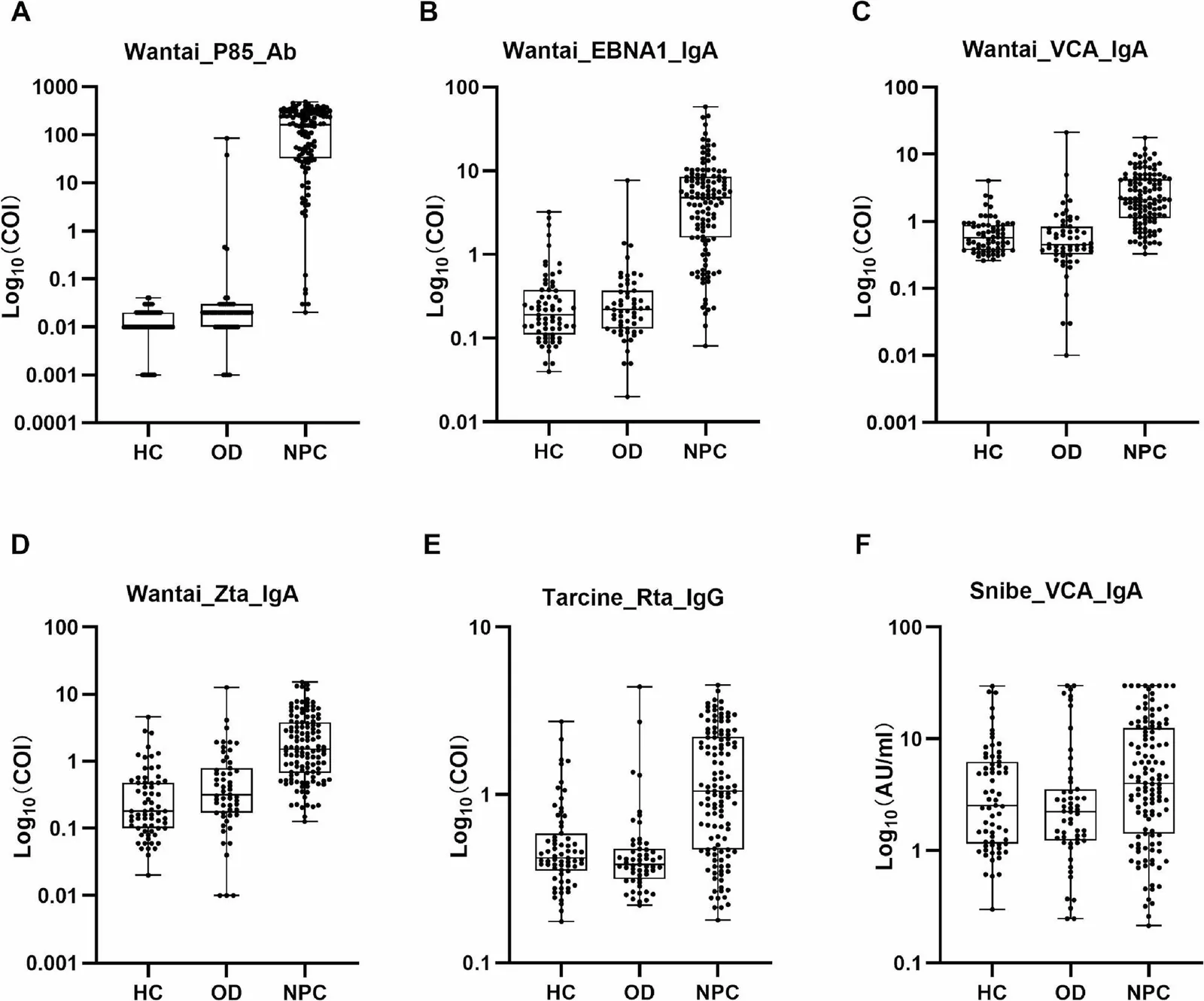
Distribution of EBV antibody biomarkers across healthy controls, non-NPC other disease patients and NPC patients. **A** Wantai_P85_Ab; **B** Wantai_EBNA1_IgA; **C** Wantai_VCA_IgA; **D** Wantai_Zta_IgA; **E** Tarcine_Rta_IgG; **F** Snibe_VCA_IgA. Box-and-whisker plots were utilized to represent the distribution of continuous variables. In these plots, the center line represents the median, the lower and upper limits of the box correspond to the first (25th percentile) and third (75th percentile) quartiles, respectively. The whiskers extend to the minimum and maximum data points within 1.5 times the IQR from the lower and upper quartiles. Data points beyond the whiskers are considered potential outliers and are plotted individually. HC, healthy controls; OD, other diseases; NPC, Nasopharyngeal Carcinoma

Among the six individual biomarkers evaluated in the post-matching cohort, Wantai_P85_Ab demonstrated the highest overall diagnostic accuracy, with an AUC of 0.989 (95% CI: 0.966–0.998), statistically higher than that of the other five individual biomarkers (Table 1; Fig. 1). Wantai_P85_Ab achieved a sensitivity of 94.26% (95% CI: 88.63–97.19) and a specificity of 98.36% (95% CI: 94.22–99.55), which shows the highest performance among the evaluated individual biomarkers (Table 1). Notable, Wantai_EBNA1_IgA, whose specificity of 93.44% (95% CI: 87.59–96.64) did not differ significantly from that of Wantai_P85_Ab (*P* = 0.070) (Table 1). The diagnosis performance was consistent with the distribution across groups for each biomarker, which is illustrated in Fig. 2.

**Table 1:** The performance of EBV antibody biomarkers after propensity score matching

Biomarker	AUC (95%CI)	*P* value	Sensitivity% (95%CI)	*P* value	Specificity% (95%CI)	*P* value
Wantai_P85_Ab	0.989 (0.966–0.998)	Ref	94.26 (88.63–97.19)	Ref	98.36 (94.22–99.55)	Ref
Wantai_EBNA1_IgA	0.942 (0.904–0.967)	< 0.001	81.15 (73.3–87.1)	0.002*	93.44 (87.59–96.64)	0.070
Wantai_VCA_IgA	0.874 (0.826–0.913)	< 0.001	77.87 (69.72–84.32)	< 0.001*	82.79 (75.12–88.46)	< 0.001*
Wantai_Zta_IgA	0.847 (0.796–0.890)	< 0.001	62.3 (53.44–70.4)	< 0.001*	83.61 (76.03–89.13)	< 0.001*
Tarcine_Rta_IgG	0.774 (0.716–0.824)	< 0.001	53.28 (44.46–61.9)	< 0.001*	90.16 (83.59–94.28)	0.006*
Snibe_VCA_IgA	0.601 (0.536–0.663)	< 0.001	50.0 (41.26–58.74)	< 0.001*	66.39 (57.62–74.16)	< 0.001*
Wantai_P85_Ab+Wantai_EBNA1_IgA (OR)^#^	/	/	97.54 (93.02–99.16)	0.125	92.62 (86.57–96.07)	0.016*
Wantai_P85_Ab+Wantai_VCA_IgA (OR)^#^	/	/	98.36 (94.22–99.55)	0.063	82.79 (75.12–88.46)	< 0.001
Wantai_EBNA1_IgA+Wantai_VCA_IgA (OR)^#^	/	/	94.26 (88.63–97.19)	1.000	78.69 (70.60-85.02)	< 0.001
Wantai_P85_Ab+Wantai_EBNA1_IgA+Wantai_VCA_IgA (OR)^#^	/	/	98.36 (94.22–99.55)	0.063	78.69 (70.60-85.02)	< 0.001

^#^The “OR” rule defines a positive combined test when either individual marker is positive

The 95% confidence interval of AUC was calculated using the Binomial exact method. The DeLong test was used for comparing AUCs of different markers. The 95% confidence intervals of sensitivity and specificity were calculated using the Wilson score method. The McNemar exact test (exact binomial test) was used to compare the diagnostic performance of paired markers. *P value < 0.05 was considered statistically significant

In the original unmatched cohort (*n* = 307; 122 NPC, 185 non-NPC), the specificity estimates of all six biomarkers closely approximated those obtained after propensity score matching, with only marginal differences (Table S2 vs. Table 1): Wantai_P85-Ab, 97.84% (95% CI: 94.57–99.16) vs. 98.36% (95% CI: 94.22–99.55); Wantai_EBNA1-IgA, 94.59% (95% CI: 90.34–97.04) vs. 93.44% (95% CI: 87.59–96.64); Wantai_VCA-IgA, 83.78% (95% CI: 77.79–88.40) vs. 82.79% (95% CI: 75.12–88.46); Wantai_Zta-IgA, 84.86% (95% CI: 79.00-89.32) vs. 83.61% (95% CI: 76.03–89.13); Tarcine_Rta-IgG, 88.65% (95% CI: 83.27–92.45) vs. 90.16% (95% CI: 83.59–94.28); Snibe_VCA-IgA, 67.03% (95% CI: 59.97–73.39) vs. 66.39% (95% CI: 57.62–74.16). This high concordance indicates that demographic confounding had negligible impact on biomarker performance and that propensity score matching did not materially alter the core findings.

### Complementary diagnostic value of EBV antibody biomarkers

**Fig. 3:**
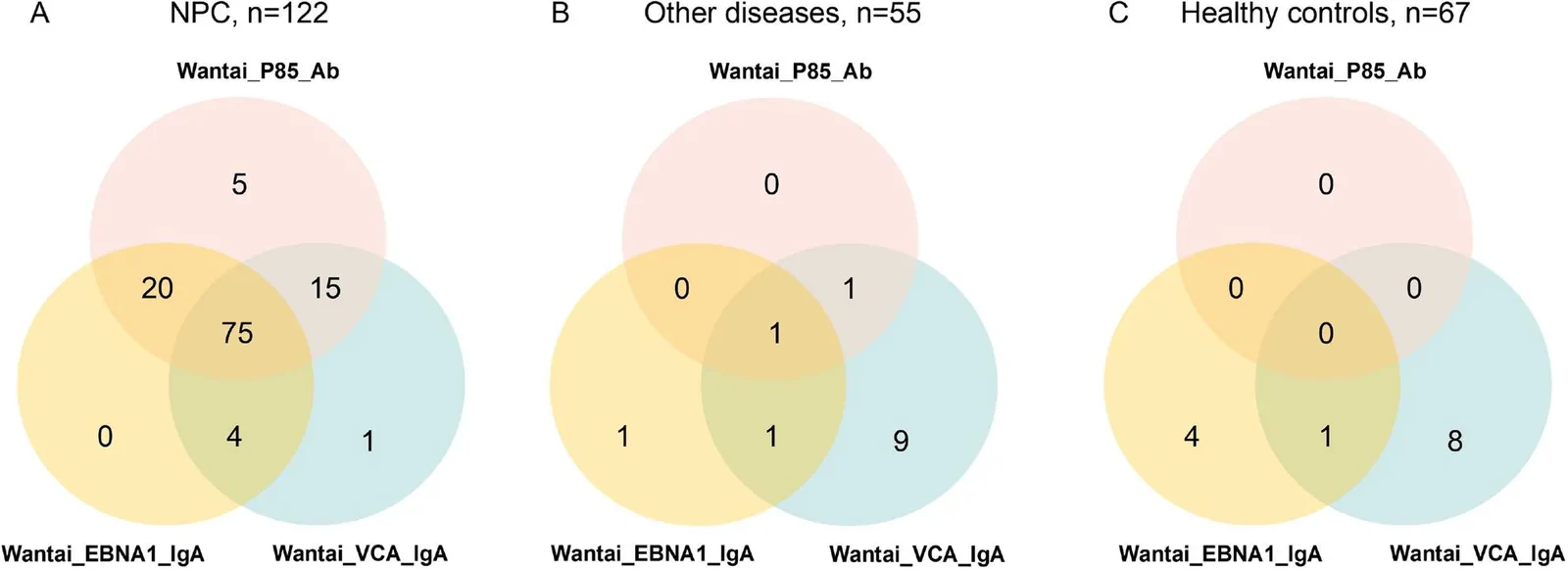
Venn diagrams for overlaps of three Wantai biomarkers in propensity score-matched groups. **A** NPC, (**B**) Other diseases, (**C**) Healthy controls. NPC, Nasopharyngeal Carcinoma

To explore whether combining biomarkers could further improve diagnostic performance, we additionally evaluated parallel testing combinations (“OR” rule) using the top three individual markers (Table 1). The combination of Wantai_P85_Ab and Wantai_EBNA1_IgA yielded a sensitivity of 97.54% (95% CI: 93.02–99.16) and a specificity of 92.62% (95% CI: 86.57–96.07), representing a modest yet clinically meaningful 3.28% absolute rise in sensitivity (from 94.26%) relative to Wantai_P85_Ab alone with a statistically significant drop in specificity (from 98.36%, *P* = 0.0156). Specifically, the combined of Wantai_P85_Ab with Wantai_EBNA1_IgA detected 4 extra NPC cases compared with using Wantai_P85_Ab alone, but at the cost of 2 extra false positives in the non-NPC other disease group and 5 extra false positives in the healthy control group (Fig. 3).

Given that EBNA1_IgA and VCA_IgA represent the current standard serological panel for NPC screening, we further investigated the complementary value of Wantai_P85_Ab to these two traditional biomarkers (Fig. 3). In the NPC cohort, 75 patients (61.5%) exhibited positive in all three biomarkers. Notably, Wantai_P85_Ab identified five cases missed by both EBNA1_IgA and VCA_IgA. Consequently, 120 of the 122 NPC patients (98.4%) were successfully detected by at least one of the three markers. Among non-NPC participants, Wantai_VCA_IgA was the predominant source of false-positive signals, accounting for 9 exclusive false-positive cases in the other disease group and 8 in the healthy control group. Regarding Wantai_P85_Ab, while 2 false-positive cases were detected in the other disease group together with EBNA1_IgA, no additional false-positive cases were introduced by Wantai_P85_Ab as a single positive marker. Collectively, these findings indicate that Wantai_P85_Ab enhances sensitivity by capturing NPC cases overlooked by traditional markers, without increasing the false-positive burden.

### Subgroup analyses by control type and tumor stage

To further characterize the diagnostic utility of the EBV antibody biomarkers, we examined their performance separately against two clinically distinct control populations in the pre-matching cohort to maximize the utilization of samples. Wantai_P85_Ab demonstrated the strongest discriminatory performance in both the HC and OD groups, achieving an AUC of 0.994 (95% CI: 0.973-1.000) with a specificity of 100.00% (95% CI: 96.26–100.00) against HCs, and an AUC of 0.982 (95% CI: 0.953–0.995) with a specificity of 95.35% (95% CI: 88.64–98.18) against ODs. Except Tarcine_Rta-IgG and Snibe_VCA-IgA, the specificities of the following biomarkers were all lower in the OD group than in the HC group: Wantai_P85_Ab (95.35% vs. 100.00%), Wantai_EBNA1_IgA (94.19% vs. 94.95%), Wantai_VCA_IgA (81.40% vs. 85.86%), and Wantai_Zta-IgA (76.74% vs. 91.92%) (Table 2). Specifically, among 73 non-NPC subjects with benign diseases, Wantai_P85_Ab tested false-positive in only 2 subjects, whereas Wantai_EBNA1_IgA, Wantai_VCA_IgA, Wantai_Zta_IgA, Tarcine_Rta_IgG, and Snibe_VCA_IgA tested false-positive in 3, 10, 14, 5, and 17 subjects, respectively. Among 24 non-NPC subjects with malignant tumors, the corresponding numbers were only 2 for Wantai_P85_Ab, and 4, 8, 7, 4, and 7 for the remaining markers, respectively (Supplementary Table S3).

**Table 2:** Differential diagnostic performance of EBV antibody biomarkers against healthy controls and other disease patients (pre-matching cohort)

Biomarker	NPC(*N* = 122) vs. HC(*N* = 99)		NPC(*N* = 122) vs. OD(*N* = 86)	
	AUC(95%CI)	Specificity% (95%CI)	AUC(95%CI)	Specificity% (95%CI)
Wantai_P85_Ab	0.994(0.973-1.000)	100.00(96.26–100.00)	0.982(0.953–0.995)	95.35 (88.64–98.18)
Wantai_EBNA1_IgA	0.949(0.911–0.974)	94.95(88.72–97.82)	0.940(0.898–0.968)	94.19 (87.10-97.49)
Wantai_VCA_IgA	0.880(0.830–0.920)	85.86(77.65–91.39)	0.875(0.822–0.917)	81.40 (71.89–88.21)
Wantai_Zta_IgA	0.906(0.859–0.941)	91.92(84.86–95.85)	0.793(0.731–0.846)	76.74 (66.79–84.41)
Tarcine_Rta_IgG	0.748(0.685–0.804)	85.86(77.65–91.39)	0.783(0.720–0.837)	91.86 (84.14-96.00)
Snibe_VCA_IgA	0.587(0.519–0.653)	60.61(50.76–69.66)	0.628(0.558–0.694)	74.42 (64.29–82.46)

*HC* Healthy control, *OD* Other diseases, *NPC* Nasopharyngeal Carcinoma

We further explored the sensitivity stratified by stage (early-stage, Stages I-II; advanced-stage, Stages III-IV) across the NPC cohort. Wantai_P85-Ab exhibited a sensitivity of 95.00% (95% CI: 76.39–99.11) among early-stage cases (*n* = 20) and 94.12% (95% CI: 87.76–97.28) among advanced-stage cases (*n* = 102). Among the remaining five biomarkers, Wantai_VCA_IgA, Tarcine_Rta_IgG and Snibe_VCA_IgA demonstrated elevated sensitivity in advanced NPC, whereas Wantai_EBNA1_IgA and Wantai_Zta_IgA showed higher sensitivity in early-stage NPC. Collectively, Wantai_P85_Ab delivered consistent stage-specific sensitivity when compared with the other five EBV antibody markers (Supplementary Table S4).

## Discussion

This prospective cohort used PSM to balance baseline confounders and conduct head-to-head comparisons of six EBV serological biomarkers. P85-Ab yielded significantly superior diagnostic performance relative to conventional VCA-IgA and EBNA1-IgA, with consistently high sensitivity and specificity for discriminating HC and non-NPC participants. Although differences in study populations and cutoff value among studies, our findings corroborate prior large-scale validations of P85-Ab, which consistently demonstrated superior performance over conventional markers in both screening and clinical settings [16, 19–24].

Further concordance analysis within NPC cohort revealed asymmetric complementary role between Wantai_P85-Ab and Wantai_EBNA1-IgA: Wantai_P85-Ab successfully identified 20 cases missed by Wantai_EBNA1-IgA, whereas Wantai_EBNA1-IgA rescued only 4 cases missed by Wantai_P85-Ab, which accounts for the limited sensitivity gain when combining Wantai_EBNA1-IgA with Wantai_P85-Ab compared with Wantai_P85-Ab alone. We adopted the OR parallel testing strategy in the present study to elevate diagnostic sensitivity, while previous researches have utilized the AND serial testing rule or regression-based scoring models [16]. The choice among these combination strategies largely reflects a trade-off between the clinical priority of maximizing sensitivity versus specificity and practical ease of implementation. In summary, P85-Ab confers significant complementary diagnostic value over conventional serological biomarkers regardless of the combination strategy applied.

Four false-positive Wantai_P85-Ab results were detected in patients with non-NPC other diseases, comprising two benign conditions and two EBV-associated malignancies. In the patient with chronic sinusitis and prior EBV infection, the positive signal likely reflects nonspecific polyclonal B-cell hyperactivation within inflamed sinonasal mucosa [28]. For the two malignancies-primary parotid lymphoepithelial carcinoma and nasal cavity lymphoepithelial carcinoma, both of them belong to the EBV-associated lymphoepithelial carcinoma spectrum, sharing undifferentiated histology and dense lymphoid stroma, and both are positive for EBER by in situ hybridization; this positivity reflects the anti-EBV immune responses elicited by the tumors themselves [29–31]. These cases illustrate a fundamental limitation of serological antibody biomarkers, which only indicate systemic immune status against EBV rather than the anatomical location of lesions. Consequently, P85-Ab can be used as a screening and triage tool whereby positive results trigger nasopharyngoscopy and/or magnetic resonance imaging while definitive diagnosis depends on pathological examination. Notably, Tarcine_Rta-IgG and Snibe_VCA-IgA paradoxically exhibited higher AUC and specificity against ODs than HCs. These unexpected findings may be attributable to the high baseline seropositivity of IgG-class antibodies among healthy EBV carriers in our cohort, different antibody distribution pattern between the OD and HC groups, and potential effects of cutoff settings on specificity estimates.

We observed reduced Wantai EBNA1-IgA sensitivity in advanced versus early NPC, consistent with multiple cohort studies [24, 32]. Mechanistically, early robust anti-EBNA1 IgA responses are blunted at advanced stages via EBNA1-mediated Treg recruitment and potential B-cell suppression [33–35]. Zta-IgA staging correlations remain conflicting across existing literature [36, 37]. However, P85-Ab exhibited comparable sensitivity for early and advanced NPC in our cohort, consistent with two large‑scale studies [23, 24]. Given the small number of early-stage cases in present study, this staging sensitivity data should be interpreted cautiously and require validation in larger cohorts.

This study has several limitations. First, as a single-center investigation conducted in a high-incidence region of Southern China, the generalizability of our performance estimates requires external validation. Second, the limited number of early‑stage NPC cases precludes definitive conclusions. Third, the case-control design does not fully reflect the test performance in a real-world. Finally, we did not systematically exclude participants who received anti-tumor treatment more than three years before enrollment, and this omission may have introduced modest bias into analyses of serological biomarkers. Large-scale multicenter prospective trials covering heterogeneous populations with sufficient early-stage NPC patients and consecutive symptomatic suspected individuals are needed to validate our results and clarify the practical clinical value of P85-Ab in routine clinical practice.

## Conclusions

In conclusion, this prospective case-control study demonstrates that the Wantai_P85-Ab exhibits significantly higher diagnostic accuracy than conventional EBV serological markers for distinguishing NPC from non-NPC individuals. Wantai_P85-Ab achieves high sensitivity and specificity as a single biomarker and provides robust specificity against a spectrum of non-NPC conditions. These data indicate that P85-Ab represents a superior single serological marker, supporting the potential application of P85-Ab as a primary serological biomarker for NPC screening and auxiliary diagnosis in endemic regions.

## Supplementary Information

Supplementary Material 1.

Supplementary Material 2.

Supplementary Material 3.

Supplementary Material 4.

## Data Availability

The datasets used and/or analyzed during the current study are available from the corresponding author on reasonable request.

## Abbreviations

NPC: Nasopharyngeal carcinoma
EBV: Epstein-Barr virus
VCA-IgA: Viral capsid antigen-IgA
EBNA1-IgA: Epstein-Barr nuclear antigen 1-IgA
EA-IgA: early antigen-IgA
PSM: propensity score matching
ROC: receiver operating characteristic
AUC: area under the curve
CI: confidence interval
HC: healthy control
OD: other disease
ELISA: enzyme-linked immunosorbent assay
CMIA: Chemiluminescent microparticle immunoassay
PPV: positive predictive value
COI: cut-off index
ENT: Otorhinolaryngology
